# Alprazolam Prompts HIV-1 Transcriptional Reactivation and Enhances CTL Response Through RUNX1 Inhibition and STAT5 Activation

**DOI:** 10.3389/fneur.2021.663793

**Published:** 2021-07-22

**Authors:** Angel Lin, Weam Othman Elbezanti, Alexis Schirling, Adel Ahmed, Rachel Van Duyne, Simon Cocklin, Zachary Klase

**Affiliations:** ^1^Department of Biological Sciences, University of the Sciences, Philadelphia, PA, United States; ^2^Department of Pharmacology and Physiology, Drexel University College of Medicine, Philadelphia, PA, United States; ^3^Center for Cellular Immunotherapies, University of Pennsylvania, Philadelphia, PA, United States; ^4^HIV-1 Dynamics and Replication Program, National Cancer Institute, Frederick, MD, United States; ^5^Department of Biochemistry and Molecular Biology, Drexel University College of Medicine, Philadelphia, PA, United States; ^6^Center for Neuroimmunology and CNS Therapeutics, Institute of Molecular Medicine and Infectious Diseases, Drexel University College of Medicine, Philadelphia, PA, United States

**Keywords:** HIV-1, latency, alprazolam, stat5, latency reversing agent, runx1

## Abstract

The HIV-1 pandemic is a significant challenge to the field of medicine. Despite advancements in antiretroviral (ART) development, 38 million people worldwide still live with this disease without a cure. A significant barrier to the eradication of HIV-1 lies in the persistently latent pool that establishes early in the infection. The “shock and kill” strategy relies on the discovery of a latency-reversing agent (LRA) that can robustly reactivate the latent pool and not limit immune clearance. We have found that a benzodiazepine (BDZ), that is commonly prescribed for panic and anxiety disorder, to be an ideal candidate for latency reversal. The BDZ Alprazolam functions as an inhibitor of the transcription factor RUNX1, which negatively regulates HIV-1 transcription. In addition to the displacement of RUNX1 from the HIV-1 5′LTR, Alprazolam potentiates the activation of STAT5 and its recruitment to the viral promoter. The activation of STAT5 in cytotoxic T cells may enable immune activation which is independent of the IL-2 receptor. These findings have significance for the potential use of Alprazolam in a curative strategy and to addressing the neuroinflammation associated with neuroHIV-1.

## Introduction

HIV-1, the causative agent of AIDS, is a retrovirus that has infected ~38 million people worldwide (1). While the advent of ART therapy has transformed the pandemic from a severe and acute condition to a chronic and manageable one, there is currently no cure for the disease due to the persistence of an HIV-1 latent reservoir (2). Integration of the HIV-1 viral DNA into host chromatin is an irreversible step in the HIV-1 life cycle, after which the activity of HIV-1 transcription is dependent on both viral and host transcription factors (3). One of the main targets of HIV-1 is CD4+ T cells. After infection, the vast majority of these target cells support replication of the virus. During this productive infection, the 5'long terminal repeat (LTR) of the HIV-1 genome acts as an inducible promoter within the host chromatin to drive viral transcription (4, 5). In a small portion of infected cells, HIV-1 remains non-productive and transcriptionally silent, therefore the viral genome persists stably in the host chromatin, and latent transcription allows the infected cell to dodge immune surveillance and its cytopathic fate (4, 6–8).

However, the condition of latency is reversible. With the correct stimulation, latent HIV-1 can be reactivated. A strategy termed “Shock and Kill” aims to purge the latent reservoir by reactivating non replicating viral genomes and resubmit the infected cells to immune clearance (9–11). One of the main obstacles in this strategy is to find chemical stimuli that not only reactivate latent virus efficiently but also promote immune clearance. While many latency reversal agents (LRAs) from different classes have been tested in laboratory settings and clinical trials, they fall short of reducing the size of the latent reservoir due to deficiencies in reactivating potential and prompting proper immune response (11–14). One example is the widely tested FDA-approved drug- Vorinostat (SAHA). Originally developed for cancer treatment as a histone deacetylase (HDAC) inhibitor, it was found to reactive latent HIV-1 transcription. However, studies suggest that HDAC inhibitors negatively impact CTL response (15–17). In short, new stimuli for latency reversal are needed and the purpose of this study is to explore a promising host candidate, RUNX1, as a target to switch viral transcription back on. The Runt related transcription factor 1 (RUNX1) is a critical host factor required for permanent silencing of CD4 in maturing CD8+ T cells (18, 19). The RUNX1 protein contains a DNA binding domain, forms a heterodimer with binding partner CBF-β to efficiently bind to DNA and regulates transcription by recruiting additional transcription factors (20). RUNX1 functions as a platform to recruit other transcription factors that have an effect on transcription. Therefore, it can serve as both an activating and a repressing factor (21–23). RUNX1 binding to the consensus sites within the CD4 silencer region is crucial for T cell differentiation through the recruitment and binding of many additional transcription factors such as HDACs and histone methyltransferase (HMT) (24, 25). RUNX1 has also been shown to bind to positive transcription elongation factor (p-TEFb), which allows RUNX1 to contribute to CD4 silencing and may facilitate HIV-1 transcriptional silencing (26–28).

The HIV-1 LTR contains a RUNX1 binding site and the binding of RUNX1 to the HIV-1 LTR suppresses HIV-1 transcription (29, 30). Using Ro5-3335, a benzodiazepine (BDZ) compound known to interfere with RUNX1 and CBF-β function, HIV-1 transcription can be moderately reactivated (29, 30). However, since Ro5-3335 in addition to inhibiting RUNX binding also inhibits Tat (31), an important viral protein that drives transcription, it is not an ideal candidate for the shock and kills strategy to reverse HIV-1 latency. The search for an ideal RUNX1 inhibitor and LRA led to the use of another BDZ compound, Alprazolam. Alprazolam was found to robustly reactivate latent HIV-1 transcription without negatively impact Tat function (29, 30). We speculated that Alprazolam might interact with RUNX1 in a similar fashion as Ro5-3335, however, the detailed mechanism was unknown.

Benzodiazepines (BDZs) such as diazepam (Valium) and Alprazolam (Xanax), are effective anxiolytic (anti-anxiety) agents approved by the FDA for the treatment of panic and anxiety disorders. This effect is the result of the ability of BDZs to positively allosterically regulate the gamma-aminobutyric acid (GABA)-A receptor in the central nervous system (CNS) (32). BDZs have well-described pharmacokinetics and penetrate the blood-brain barrier (32, 33), making them an attractive therapy to address issues in the CNS. Persistent HIV-1 infection of CNS reservoirs drives a spectrum of neuropathologic, behavioral, and cognitive effects (34–43). Even with effective ART therapy, these neuropathologies are apparent in HIV-1 infected individuals (44, 45). The adverse neurologic outcomes associated with HIV-1 infection are thought to be primarily driven by chronic neuroinflammation (46–48). Understanding the mechanisms by which BDZs affect HIV-1 transcription and any associated effect on immune function may allow us to design therapies to address the latent reservoir and immune dysfunction in both the periphery and the CNS.

This study presents evidence that Alprazolam is working as a *bona fide* RUNX1 inhibitor and drives Signal Transducer and activator 5 (STAT5) recruitment to the HIV-1 LTR driving latency reactivation. In the CNS, STAT5 is expressed in the hypothalamic arcuate nucleus (ARC), dopaminergic and somatostatin neurons (49–51) and preferentially activated by granulocyte-macrophage colony-stimulating factor (GM-CSF). It has been shown that HIV-1 infection negatively impacts the activation level of both STAT5 and GM-CSF and therefore may impair macrophage function (52). GM-CSF has been tested as an adjuvant of ART in clinical studies and demonstrated to improve host defense and immune outcomes such as increased CD4+ T cell count and decreased plasma HIV-1 RNA detected in HIV-1 patients. The mechanism behind such effect is unclear but may be associated with STAT5 activation (53, 54).

The cytotoxic T cells (CD8+ T cells) are essential in the recognition of virally infected cells and foreign antigens. IL-2 is responsible for CD8+ T cell activation and the differentiation into potent effector cells to elicit rapid expansion and eradicate infections (55). STAT5 is activated by a wide variety of cytokines and growth factors, including IL-2 and GM-CSF, through phosphorylation. STAT5 is a 90kDA protein encoded by two closely related genes (STAT5a and STAT5b), located on human chromosome 17 (56). The activation by phosphorylation is targeted to the tyrosine residue 694 and 699 on STAT5A and STAT5B, respectively, by the receptor-associated Janus family tyrosine kinase (Jak) (57). Activated STAT5 then dimerize as a STAT5A/B heterodimer, translocate to the nucleus, and induce gene transcription that is crucial to T cell survival, proliferation, and cytokine production (56, 58, 59). The STAT5a/b heterodimer commonly binds to the consensus sequence TTC (T/C) N (G/A) GAA which is the interferon gamma-activated sequence (GAS) motif (60). STAT5 binding site was also found on the HIV-1 promoter (61) and we show that the activation of STAT5 via Alprazolam may potentiate IFNγ production in CD8 T cells. This study shows evidence to demonstrate that Alprazolam acts as a RUNX1 inhibitor and potential LRA that may positively impact immune response toward HIV-1 infected cells.

## Results

### BDZs That Alter RUNX1 Activity May Directly Interact With RUNX1

Our recent publication provided evidence that BDZs alter the epigenetics of the integrated HIV-1 LTR and activate viral transcription (30). The structural similarities of clinically relevant BDZs such as Alprazolam, Diazepam, and Clonazepam to the RUNX1 inhibitor Ro5-3335 suggests that these compounds also interact with RUNX1 to affect its function. Although studies have shown interactions between Ro5-3335 and RUNX1, no information is currently available on how these two molecules interact.

**Figure 1:**
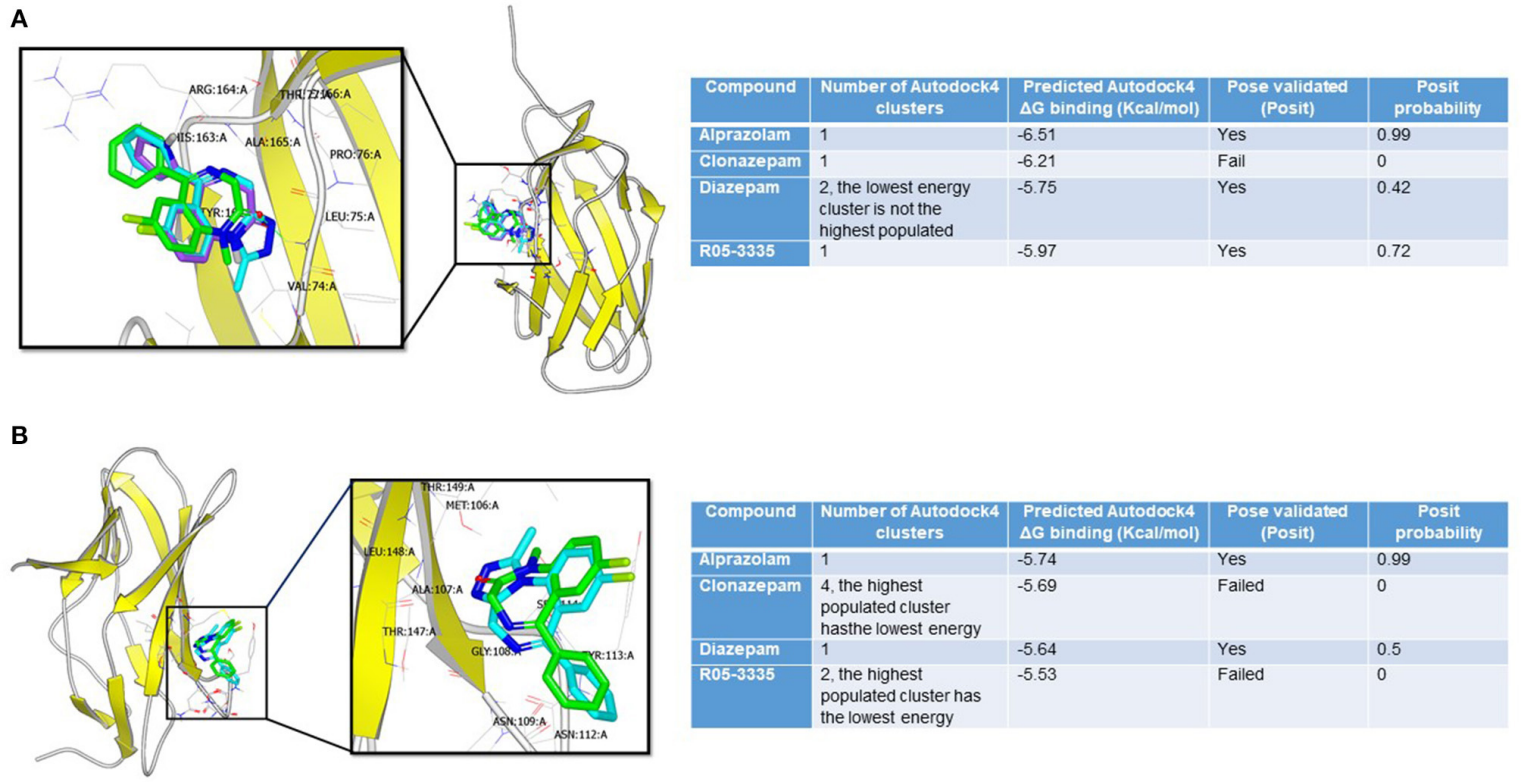
Binding modes of benzodiazepines to RUNX1. **(A)** Alprazolam (cyan), diazepam (green), and R05-355 (purple) were predicted to bind at the bound pocket. Alprazolam has the highest POSIT probability (0.99 vs. 0.42 for diazepam and 0.72 for R05-355). **(B)** Only alprazolam (cyan) and diazepam (green) were predicted to bind at the unbound-RUNX1 pocket. Alprazolam has the highest POSIT probability (0.99 for alprazolam vs. 0.5 for diazepam).

To investigate whether the structural similarities between BDZs and Ro5-3335 translates into being able to interact with RUNX1, docking studies were performed. We evaluated the potential interaction of Ro5-3335, Alprazolam, Clonazepam, and Diazepam using the docking programs Gold and Autodock. We chose to also investigate whether these compounds may prefer the liganded or unliganded forms of RUNX1. The unbound (PDB:1EAN) (Figure 1A) and the bound (PDB: 3WTS) (Figure 1B) structures of RUNX1 share an overall backbone structure but differ in the orientation of several side chains. Druggable sites were found on each structure through quick blind docking of the ligands using the two docking software to the entire protein. Subsequently, these pockets were selected for further in-depth analysis using the cluster analysis function of Autodock4 (62, 63). Only alprazolam and diazepam were predicted to bind to the unbound form (Figure 1A), while alprazolam, diazepam and Ro5-3335 were predicted to bind to the bound form (Figure 1B).

This analysis suggests that these BDZs could interact with RUNX1 and that their potential binding sites are on a face distinct from the known interaction regions for DNA and CBFβ. Additionally, these analyses suggest a second binding pocket on RUNX1 that may be engaged only by Alprazolam and diazepam, but not Ro5-3335 and clonazepam. The differential binding modes suggest that Alprazolam and diazepam may mediate other effects on RUNX1 function beyond that seen with Ro5-3335.

### Alprazolam Alters the Expression of RUNX Responsive Genes in PBMCs

**Figure 2:**
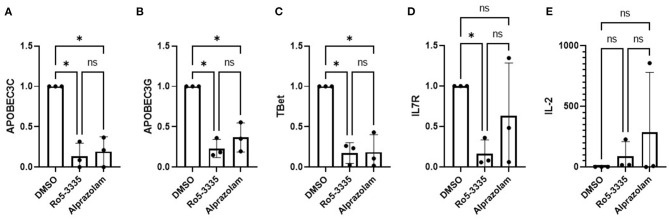
Effect of alprazolam *ex vivo* on RUNX1 responsive genes. Peripheral blood mononuclear cells (PBMC) from three HIV-1-1 positive individuals were treated with 10μM Alprazolam or Ro5-3335 for 48 h. RNA was harvested using Trizol and cDNA was synthesized by RT reaction. qPCR was performed for five known RUNX responsive genes: **(A)** APOBEC3C, **(B)** APOBEC3G, **(C)** Tbet, **(D)** IL-7R and **(E)** IL-2. Expression levels are plotted as GAPDH normalized expression with the levels in the control for each individual. Dots represent each individual data point and graphs show the average. ANOVA was used to determine statistical differences. *p < 0.05.

To determine if Alprazolam functions as a RUNX1 inhibitor we examined the expression of several RUNX responsive genes in PBMCs in response to Ro5-3335 and Alprazolam treatments (Figure 2). PBMCs from three HIV-1 patients were treated with alprazolam or Ro5-3335 for 48 h. RNA was harvested using Trizol and cDNA was synthesized by reverse transcription. qPCR was performed using primers for five known RUNX1 responsive genes: APOBEC3C, APOBEC3G, T-bet, IL7R, and IL-2 (64–70). As expected, as a RUNX1 inhibitor, Ro5-3335 significantly suppressed the expression of all the selected RUNX responsive genes except IL-2. Similarly, Alprazolam treatment resulted in a statistically significant decrease in APOBEC3C, APOBEC3G, and T-bet expression. IL-2 has been reported to be negatively regulated by RUNX1. In this assay, treatment of cells with Ro5-3335 and Alprazolam resulted in no statistically significant change in IL-2 expression. The suppression of APOBEC3C, APOBEC3G and T-bet mRNA in a manner similar to the known RUNX1 inhibitor Ro5-3335 suggests that Alprazolam inhibits RUNX1.

### Effect of Ro5-3335 and Alprazolam on IL17 Promoter-Reporter

**Figure 3:**
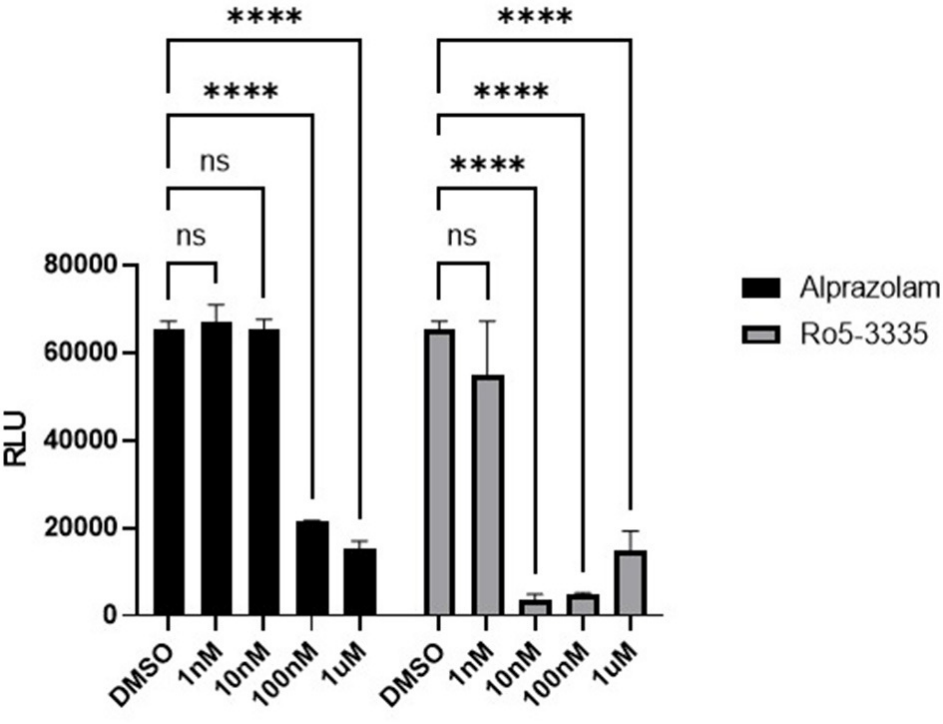
Effect of alprazolam on IL17. 293T cells were transfected with IL17-luciferase reporter plasmid and treated with Ro5-3335 or alprazolam. Luciferase activity was measured using a luciferase assay kit. Luciferase light units after treatment with RO5-3335 or alprazolam with overexpression of RUNX1. ****P ≤ 0.0001, ns = not significant.

We next sought to determine if Alprazolam could suppress RUNX1 mediated transcription in a reporter assay. RUNX1 is known to form a complex with ROR⋎t, the orphan nuclear receptor, and bind to IL17 enhancer and promoter to up-regulate IL17 expression (71, 72). We tested the ability of Ro5-3335 and Alprazolam to alter the expression of luciferase under the control of the IL17 promoter. We hypothesized that a RUNX inhibitor should be able to suppress the activity of the IL17 promoter. 293T cells were transfected with an IL17 promoter luciferase reporter plasmid and treated with Ro5-3335 and Alprazolam (Figure 3). As expected, Ro5-3335 inhibited IL17 promoter activity in a dose-dependent fashion with a maximal decrease in activity of 95.56% observed at 100 nM. Alprazolam also induced a dose-dependent decrease in luciferase activity with the greatest inhibition noted at 1μM (76% inhibition). These experiments demonstrate that alprazolam has an effect on the expression from IL17 promoter, but at higher concentrations than Ro5-3335 with IC 50 at 43.25 nM and 1.53 nM, respectively (Supplementary Figure 1).

### Benzodiazepine Activation of the LTR Is Associated With Recruitment of STAT5 and CBP/P300

**Figure 4:**
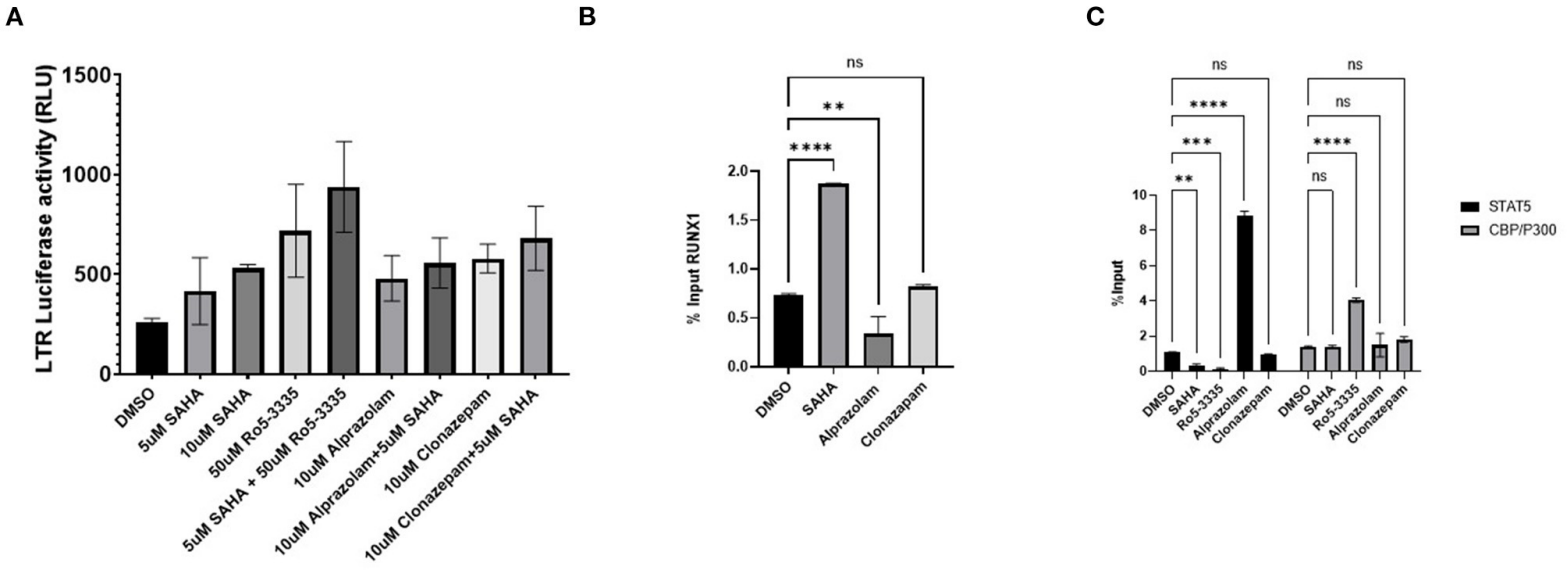
Recruitment of STAT5 and CBP/P300 to the LTR by benzodiazepines in TZMBL Cells. TZMBL cells were treated with Benzodiazepines: RO5-3335, Alprazolam and Clonazepam with or without HDACi SAHA for 48 hrs at the indicated concentrations. **(A)** Cells were lysed and LTR driven luciferase was determined. **(B)** ChIP analysis was performed to determine RUNX1 binding to the HIV-1 LTR. **(C)** ChIP analysis was performed to determine STAT5 and CBP/P300 binding to the HIV-1 LTR. **P ≤ 0.01, ***P ≤ 0.001, ****P ≤ 0.0001, ns = not significant.

Considering that Ro5-3335 reactivates HIV-1 transcription through RUNX1 inhibition, we hypothesize that Alprazolam may produce the same effect. To evaluate the latency reactivation potential of Alprazolam, TZMbl cells, a Hela derived cell line with HIV-1 LTR- driven luciferase reporter was used to measure transcriptional activation. TZMbl cells were treated with Alprazolam, or two additional BDZs: Ro5-3335 and Clonazepam at 10μM, with or without 5μM SAHA for 48 h. After which, cells were lysed and luminescence was measured. As expected, SAHA as an HDAC inhibitor increased the amount of LTR activation. All BDZs tested, including Alprazolam, Clonazepam and Ro5-3335 also activated the LTR and displayed a moderate additive effect when combined with SAHA (Figure 4A). To understand the mechanism of the BDZ-driven activation, ChIP analysis was performed. STAT5 is an important signal transactivator induced by cytokines and interleukins, and a coactivator of CBP/P300 which plays an important role in transcriptional activation. Previous studies have revealed a role for RUNX1 in suppressing STAT5 activity due to a >50% chance of sharing a binding motif (73, 74). ChIP revealed as RUNX1 is dislodged from the HIV-1 LTR by Alprazolam (Figure 4B), a significant increase in the amount of STAT5 and CBP/P300 recruited to the HIV-1 LTR when HIV-1 LTR transcription was reactivated by Alprazolam and Ro5-3335, respectively. While the treatment with Clonazepam does not affect STAT5 nor CBP/P300 recruitment, SAHA and Ro5-3335 treatment negatively impacted STAT5 recruitment (Figure 4C). This is in agreement with our recently published findings that show recruitment of STAT5 and CBP/P300 to the HIV-1 LTR in the presence of alprazolam, but not clonazepam nor Ro5-3335 (30).

### The Loss of RUNX1 Binding to the HIV-1 LTR Also Increases the Recruitment of STAT5 and CBP/P300

**Figure 5:**
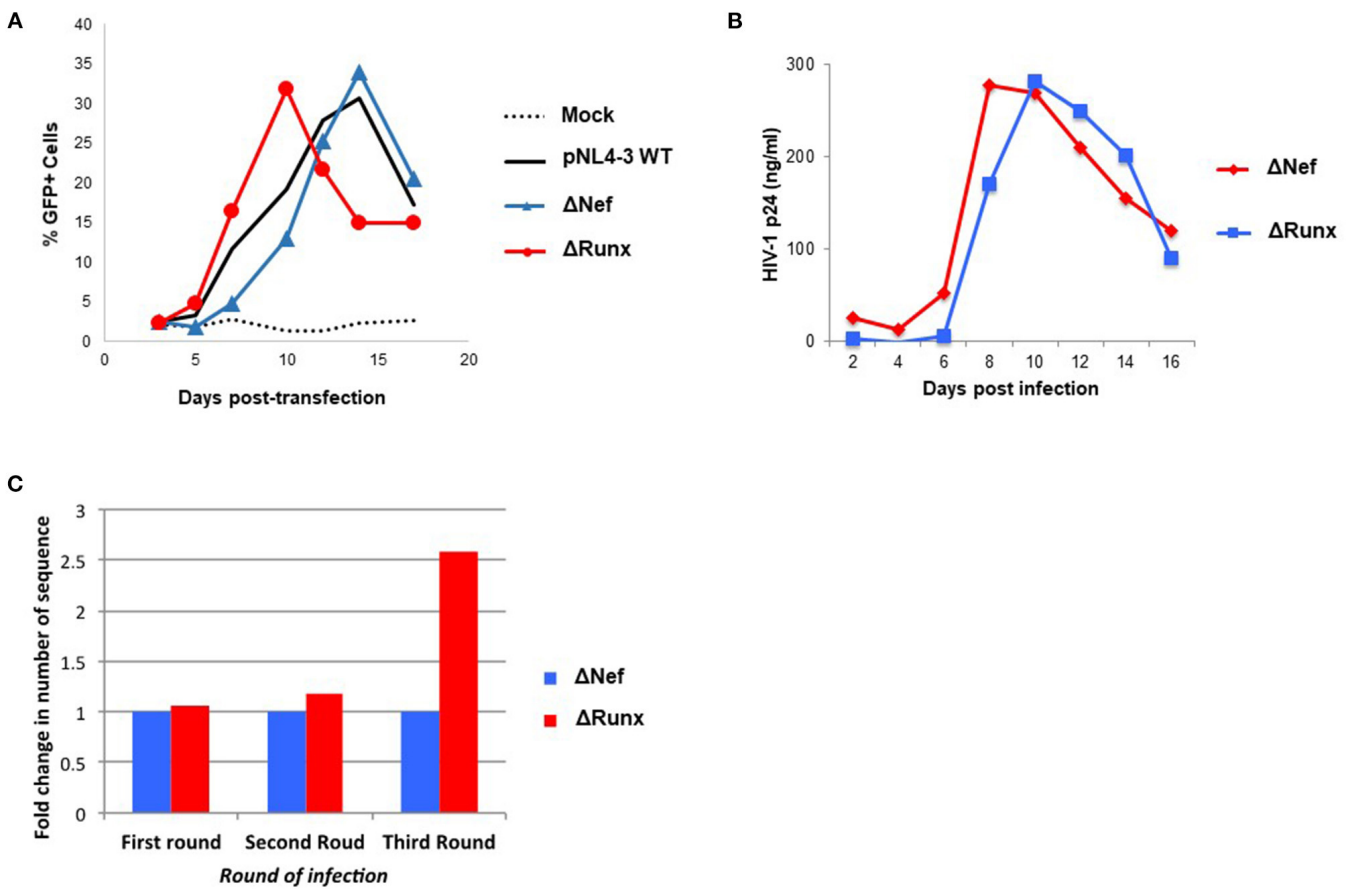
Deletion of the RUNX1 binding site from the HIV-1 LTR enhances replication kinetics relative to WT. **(A)** J-LTR-GFP cells were transfected with pNL4-3 WT, ΔNef, ΔRUNX molecular clones and replication was monitored by measuring the percentage of GFP positive cells in the culture every 2-3 days post-transfection. **(B)** Jurkat cells were infected with equal nanogram quantities of viral stocks for ΔNef or ΔRUNX and replication was followed over time by p24 ELISA. **(C)** A competition assay was performed where J-LTR-GFP were infected with equal infectious units of the two viruses and supernatant from the peak of infection was used to infect a new round of cells. Extracellular RNA isolated from the peak of each round of infection was amplified using primers against the LTR and sequenced to be able to determine the relative abundance of each virus in each round of infection.

We next sought to determine if elimination of the RUNX1 binding site in the HIV-1 LTR would similarly increase STAT5 recruitment. To study the effect of RUNX1 binding on HIV-1 replication, an HIV-1 molecular clone that has a mutation in the RUNX1 binding site was constructed using site-directed mutagenesis. Our lab has shown that RUNX1 binds to U3 of the HIV-1 LTR (29). A point mutation in the first and second residues in the RUNX1 binding site, 55-60bp downstream of the beginning of the HIV-1 LTR (from ACCACA to CACACA), was performed as mutation of these nucleotides abrogates RUNX binding and eliminates RUNX1 effect on an HIV-1 LTR driven reporter (29). Mutations were generated in the 3′LTR of pNL4-3, as the U3 region from the 3' LTR is propagated during reverse transcription. Moreover, because the *nef* gene overlaps with the RUNX1 binding site at the 3′ LTR and nef is dispensable in the cell culture, mutations were generated in a nef-minus (Δnef) virus. The resulting proviral plasmid was designated pNL4-3 Δnef mutRUNX BS (ΔRUNX). We transfected the Δnef and ΔRUNX molecular clones into J-LTR-GFP cells and observed viral replication as measured by the accumulation of GFP+ cells over time. We observed faster replication kinetics in cells infected with the ΔRUNX compared to wildtype (WT) and ΔNef viruses, which is consistent with an increase in LTR activity upon inhibiting RUNX1 (Figure 5A). Similar results were seen when we infected Jurkat cells with Δnef or ΔRUNX virus and measured virus production in the supernatant over time (Figure 5B). To understand the effect that mutating the RUNX1 binding site has on HIV-1 viral fitness we performed a competition assay over multiple rounds of infection. J-LTR-GFP cells were infected with ΔNef and ΔRUNX viruses at a 1:1 ratio of infectious units. Cells were washed after 24 h and GFP expression was measured for 14 days. Supernatant from the peak of infection was used to infect the next round. After three rounds RNA was extracted from supernatant from the peak of each infection and RT-PCR was performed against the LTR to generate fragments for TA cloning and subsequent sequencing. The relative abundance of each virus (Δnef or ΔRUNX) was determined for each round (Figure 5C). An increase in the abundance of ΔRUNX was observed in each round (1.06-fold, 1.17-fold, and 2.5-fold). Taken together this data indicates that the ΔRUNX virus has better fitness than the Δnef control virus.

**Figure 6:**
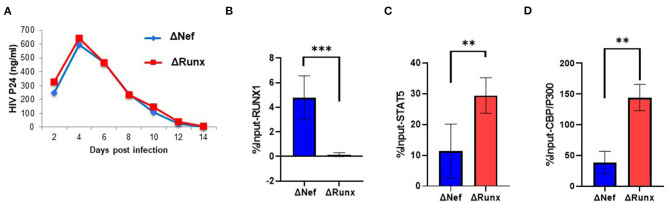
Recruitment of STAT5 and CBP/P300 to the LTR in Primary Cells with RUNX1 binding inhibition. PBMC blasts from healthy donors were infected with ΔNef or ΔRUNX. **(A)** Viral replication was followed over time by p24 ELISA. After 48 h infection, PBMC cells were fixed and prepped for CHIP analysis with IP targeting **(B)** RUNX1 **(C)** STAT5 and **(D)** CBP/P300. **P ≤ 0.01, ***P ≤ 0.001.

Treatment of cells containing an integrated HIV-1 LTR with RUNX1 inhibitors Ro5-3335 or alprazolam showed an increase in LTR associated STAT5 and associated transcription (Figure 4). To verify the effect of decreased RUNX1 binding on primary cells, PBMC from healthy donors were infected with Δnef or ΔRUNX (Figure 6A). Infected PBMCs at 48 h post-infection were fixed for ChIP-qPCR analysis. ChIP for RUNX1 confirmed that mutating the RUNX1 binding site results in loss of RUNX1 at the integrated HIV-1 LTR in primary cells infected de novo (Figure 6B). Elimination of the RUNX1 binding site increased the presence of STAT5 and subsequently CBP/P300 on the LTR (Figures 6C,D).

### Alprazolam Increases the Global Phosphorylation and Activation of STAT5

**Figure 7:**
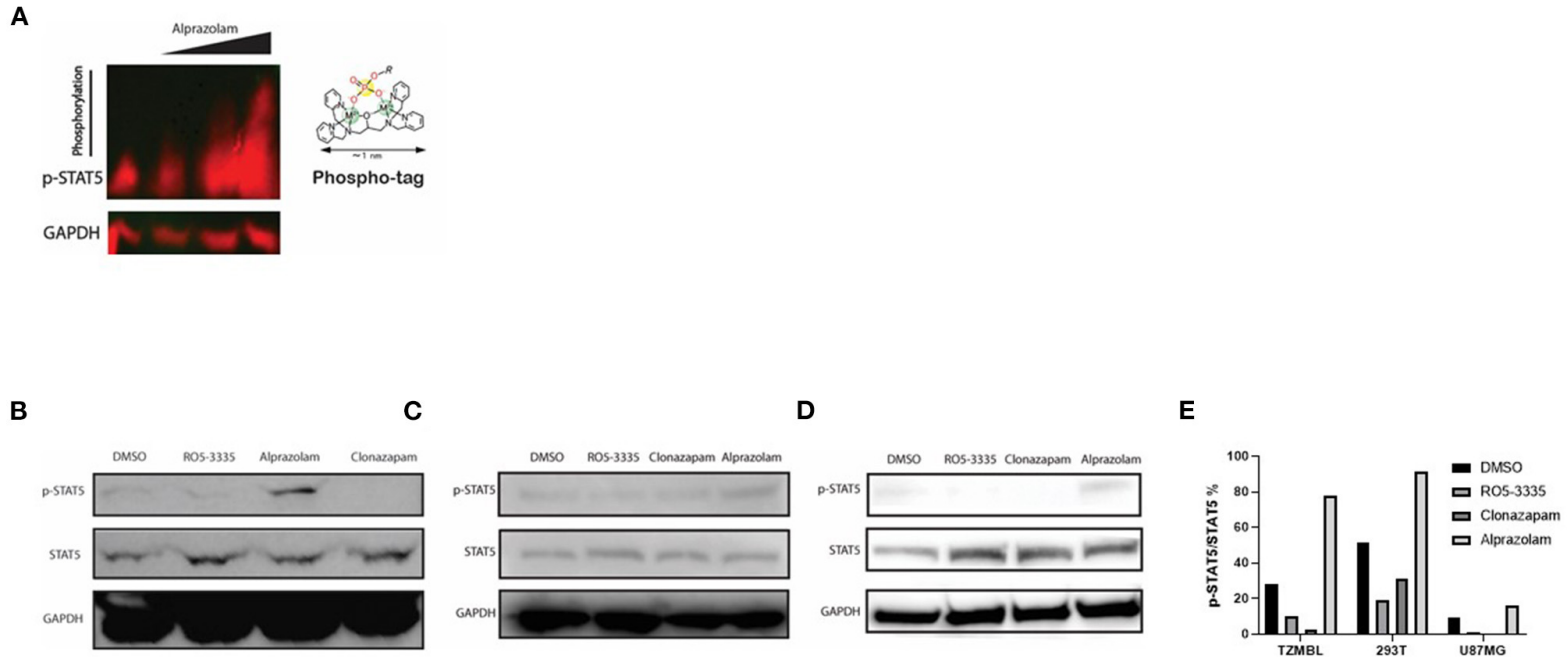
Expression of Activated STAT5 was increased when TZMBL cells were treated with Alprazolam. TZMBl **(A, B)**, HEK293T **(C)** and U87MG **(D)** cells were treated with Benzodiazepines: 50 μM RO5-3335, 10μM Alprazolam, or 10μM Clonazepam for 48 h. Cells were lysed and whole cell lysate was collected and loaded onto an SDS-PAGE Gel **(A)** Phospho-Tag SDS-PAGE was performed to evaluate the dose effect of alprazolam on STAT5 phosphorylation. From left to right, TZMBL cells were treated with DMSO, 0.1μM, 1μM and 10μM of Alprazolam. **(B–D)** Western Blot was performed to evaluate the effect of Benzodiazepines on STAT5 expression and phosphorylation **(E)** Densitometry was performed and the ratio of activated STAT5 over global STAT5 was graphed.

Alprazolam induces the recruitment of the transactivator STAT5 to the HIV-1 LTR (Figure 4C). However, it was unclear whether this effect is specific to the HIV-1 LTR or due to broad activation of STAT5 due to RUNX1 inhibition. To examine this, Phospho-Tag Gel was used to further retard the movement of phosphorylated proteins in the gel and western blotting was performed to visual STAT5 specifically. TZMbl cells were treated with Alprazolam at the concentration of 0uM, 0.1um, 1uM, and 10uM. The increase of phosphorylation on STAT5 is dose-dependent when TZMbl cells were treated with 1uM and 10uM of Alprazolam (Figure 7A). To verify phosphorylation on STAT5, TZMbl (Figure 7B), HEK293T (Figure 7C) and U87MG (Figure 7D) cells were treated with BDZs for 48 h followed by whole-cell protein extraction and western blot for STAT5 protein. It was discovered that Alprazolam does not increase the expression of unmodified STAT5, yet it does increase the amount of Y694 phosphorylated or activated STAT5. Ro5-3335 and Clonazepam, compounds that did not induce STAT5 recruitment in our ChIP assays [Figure 4 and (30)] did not induce STAT5 phosphorylation. Densitometry analysis reveals an elevation in the ratio of activated STAT5 to global STAT5 is induced by Alprazolam treatment (Figure 7E).

**Figure 8:**
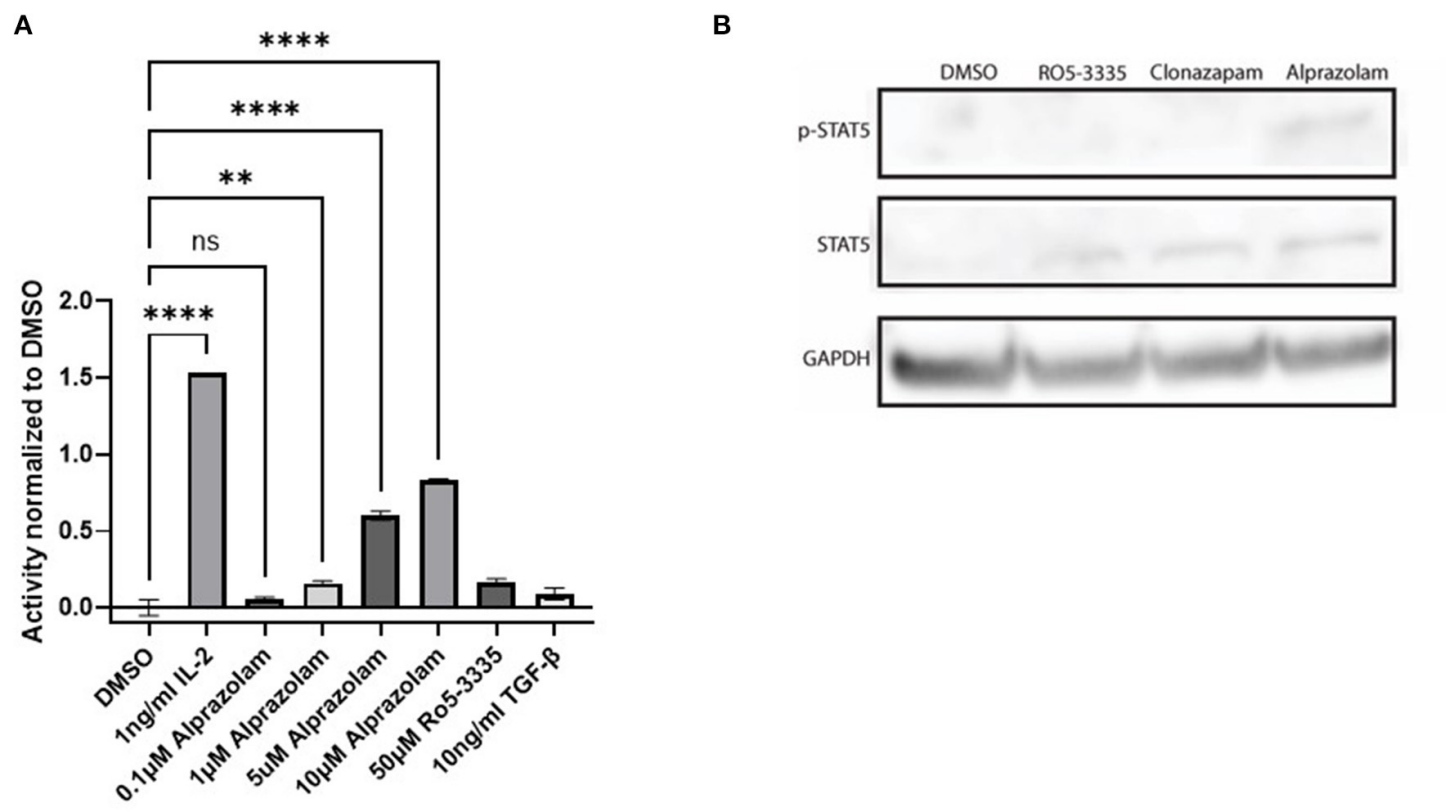
Alprazolam activates a STAT5 sensitive reporter. HEK-Blue-IL2 cells were treated with indicated doses of IL-2, alprazolam or Ro5-3335 and incubated for 48 h at 37C 5% CO2. **(A)** Extracellular alkaline phosphatase activity was then measured by colorimetric assay. Results show the average absorbance of three replicates. **P ≤ 0.01, ****P ≤ 0.0001, ns = not significant **(B)** Western blot was performed to evaluate the effect of Benzodiazepines on STAT5 expression and phosphorylation.

Tyrosine phosphorylation of STAT5 proteins enables nuclear translocation and enhances transcriptional activity. We demonstrated that Alprazolam reactivates HIV-1 transcription specifically through the recruitment of STAT5 and elevation of STAT5 phosphorylation (Figure 7). To confirm whether Alprazolam also upregulates STAT5 mediated transcriptional activity, we utilized the HEK-BLUE IL-2 reporter cell line, a HEK293 derived cell line that contains the IL-2 receptor, signaling cascade through the tyrosine kinases of the Janus family (Jak1/Jak3) and the STAT5-inducible gene- secreted embryonic alkaline phosphatase (SEAP). HEK-BLUE IL-2 cells were seeded in a 96 well plate and treated with Alprazolam ranging from 1μM to 5μM, 50μM Ro5-3,335 or the positive and negative controls IL-2 and TGF-ß, respectively, for 24 h. SEAP protein was then harvested from the cell suspension, stained using QUANTI-BLUE, and measured using the absorbance read at 650 nm. Alprazolam was shown to robustly enhance STAT5 mediated activation in a dose-dependent manner (Figure 8A). This is consistent with the ChIP data that demonstrates that although both compounds function as RUNX1 inhibitors, only Alprazolam enables the recruitment of STAT5 to the HIV-1 promoter. Western blot analysis also shows an elevation of phosphorylated STAT5 on Y694 when HEK-Blue IL-2 is treated with Alprazolam but not Ro5-3335 nor Clonazepam (Figure 8B).

### Alprazolam Positively Affects Intracellular Production of IFNγ and TNFα in HIV-1 Gag Responsive CD8+ T-Cells From People Living With HIV-1

**Figure 9:**
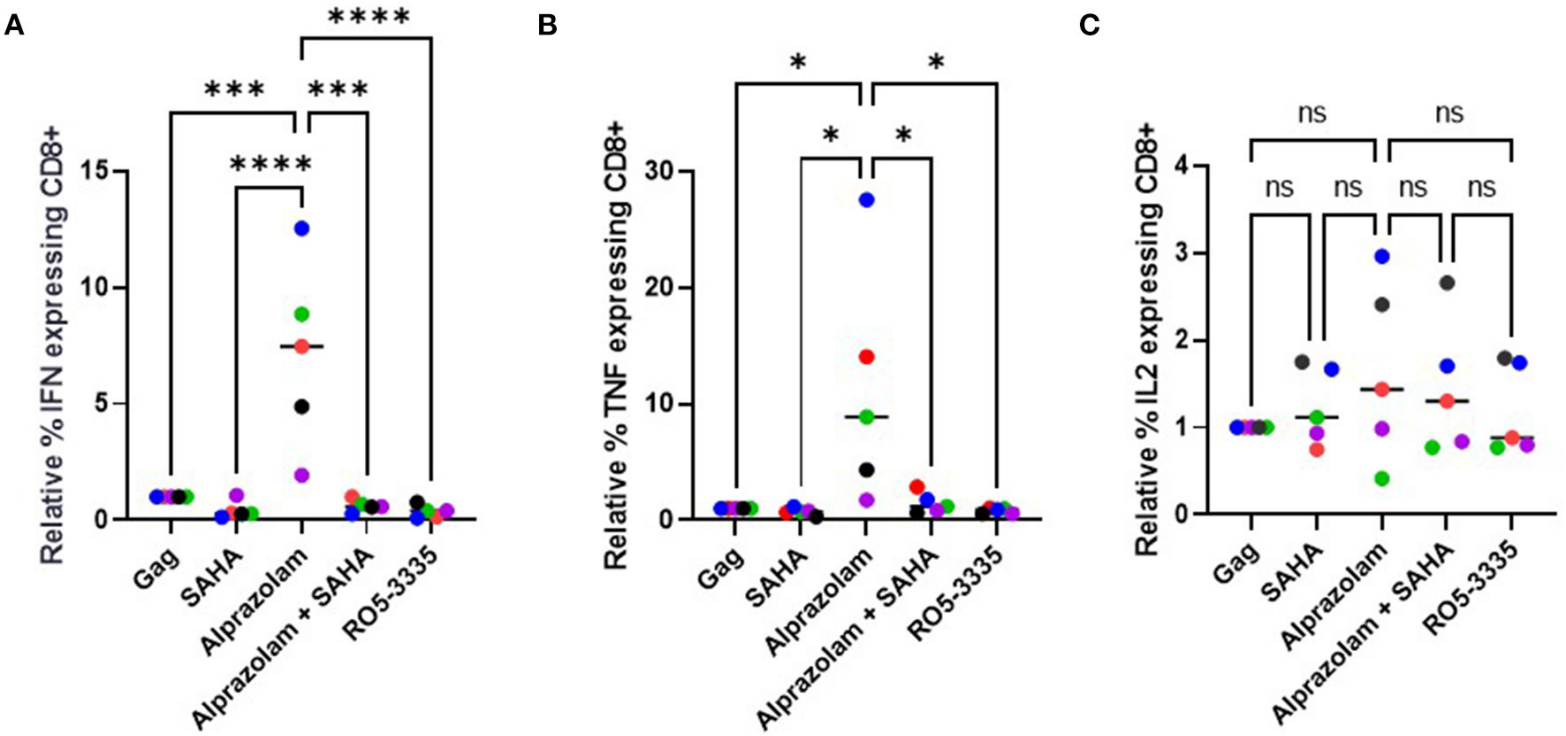
The effect of alprazolam and SAHA on Gag specific CTL response. PBMCs from 5 HIV-1 positive individuals suppressed on therapy were treated with vehicle control, 500 nM SAHA, 100 nM alprazolam, SAHA plus alprazolam or 100nM Ro5-3335 overnight and then exposed to a set overlapping Gag peptides in the presence of Brefaldin A for 6 h. Cells were stained for live/dead marker, CD3, CD4, CD8 and cytokines of interest. The number of **(A)** IFNγ, **(B)** TNFα **(C)** IL-2 expressing CD8+ T-cells for each condition is shown normalized to untreated control. *P ≤ 0.05, ***P ≤ 0.001, ****P ≤ 0.0001, ns = not significant.

Evidence shows that ART treatment, while effective in reducing viral load in a patient, also negatively impacts their CD8+ T cell response (75). The induction (or “shock”) of viral transcription is supposed to be followed by extermination of the reactivated cells by the crucial effector CTLs (76). Unfortunately, some LRA such as the HDAC inhibitors have been shown to suppress CTLs (77). STAT5 activation is critical in multiple immune functions, including T-cell response. We hypothesized that Alprazolam's ability to potentiate STAT5 activity might result in an improved response in CTLs. To test this, PBMCs from three HIV-1-infected subjects whose viral load had been suppressed on ART for >6 months were used to evaluate cytokine expression response to HIV-1 Gag peptides in the presence of 0.5 μM SAHA, 0.1 μM Alprazolam, the two in combination, or 0.1 μM Ro5-3335. In brief, PBMCs were treated overnight in RPMI supplemented with FBS and Glutamine. After treatment, cells were exposed to pooled HIV-1 Gag peptide and Golgi Stop (BD Biosciences), to prevent the export of cytokines, for 6 h before intracellular cytokine stating for IFNγ, IL-2, and TNFα was performed (Figure 9, Supplementary Figure 2). The addition of either SAHA, alprazolam or Ro5-3335 before Gag peptide treatment allowed us to examine changes in Gag induced cytokine expression in response to these drugs. Although SAHA treatment decreased the percentage of cells expressing IFNγ and TNFα in cytotoxic T cells in response to Gag in four of five subjects; the changes were not statistically significant. Alprazolam treatment caused more IFNγ and TNFα production than either SAHA, SAHA and Alprazolam combined, or Ro5-3335 treatment (Figures 9A,B). None of the treatments affected the number of cells expressing IL-2 in response to Gag (Figure 9C). Alprazolam has no effect on any of the three cytokine production in CD4 ^+^ T cells (Supplementary Figure 3).

## Discussion

CD8+ T cells are an important part of the immune response toward viral infection (78). CD8+ T cells rely on direct (cell to cell interaction) or indirect (cytokine) stimulation mediated by CD4 T cells to differentiate into effector cells (79). Despite the effectiveness of ART, HIV-1 infection has been associated with defects in cytokine production and while SAHA, a promising LRA, demonstrates reactivating potential for latent transcription, the negative impact HDACi impose on the CTL response does not align with the objective of eradicating the HIV-1 reservoir (15–17). Alprazolam has been shown to robustly reactivate latent transcription (30). Our data suggest that Alprazolam may have additional utility due to the activation and recruitment of STAT5 to the HIV-1 LTR while SAHA negatively impacts the recruitment of STAT5. STAT5 binding to the HIV-1 promoter is directly associated with transcriptional activation of the HIV-1 genome. Interestingly, Alprazolam can also activate STAT5 in non-HIV-1 infected cells, therefore, despite CD8+ T cells being a non-target to HIV-1 infection, the activation of STAT5 in CD8 cells may have allowed increased biding of STAT5 on the IFNγ promoter and jump-start cytokine production. The ability to activate CD8 T cells without increased cytokine production from CD4+ T cells is beneficial from a therapeutic standpoint since CD4 T cell's immunoregulatory function is often dysregulated and HIV-1 disease progression is associated with the loss of CD4 T cells (Supplementary Figure 3) A study has demonstrated that a subset of ART-naive patients was unable to functionally activate STAT5 in response to IL-2 stimulation on their CD8+ T cells *ex vivo* (48). The use of Alprazolam as an alternative method to activate STAT5 may potentially help with patients who are resistant to the therapy.

STAT5 activation can occur in multiple parts of the HIV-1 life cycle. Interaction between the HIV-1 viral envelope gp120 and the CD4 receptor can induce STAT5 activation and DNA binding (57). HIV-1 Nef also indirectly activates STATs (60). During viral infection, interferons (IFNs) induce transcription of interferon-stimulated genes (ISG) through the activation of STATs (80). Though STATs are crucial for antiviral and inflammatory responses, HIV-1 may have evolved to limit STAT5 activation to evade immune clearance, since it has been shown that HIV-1 infected cells do not induce high interferon levels (81) and STAT5 expression is reduced in HIV-1 patients (49). Impaired production of cytokines can be a predictor of the morality of HIV-1 patients. patients with more robust cytokine production are associated with longevity as STAT5 activation in CD8+ T Cells promotes its effector and memory development (82, 83).

It has been shown that SAHA and other HDACs impair CTL function by inhibiting IFNγ production (80). The literature suggests that Alprazolam enhances immune function (84). It has been shown that when BDZs are taken at physiological levels, they have immunoprotective effects. BDZs have also been shown to enhance the antibody response through stimulating helper T cell functions in restraint-stressed mice (85). Furthermore, Alprazolam and midazolam can decrease the adverse effect of surgical stress on the thymus and spleen in mice and maintain their cellularity (86, 87). In addition, Alprazolam protected and enhanced the immune system by increasing the activity of natural killer cells and enhancing the proliferation of lymphocytes in mice (88). Diazepam has been reported to negatively impact phagocytic activity in polymorphonuclear cells (PMN) and monocytes, triazolobenzoldiazepines like alprazolam and triazolam was shown to enhance T cell function and antibacterial activity (89). These seemingly conflicting results may be due to the ability of only a certain subclass of BDZs to bind to the second potential target site on RUNX1 (Figure 1) a prediction that correlates with modulation of STAT5 activity both on the LTR and in the whole cell.

In addition to viral transcriptional reactivation and CTL response enhancement, to achieve the goal of viral eradication, it may be important to target immune checkpoints as well. T cell exhaustion due to chronic inflammation can reduce the polyfunctionality of CD8+ T cells. It has been shown that elevated STAT5 activation in CD8+ T cells is associated with the decreased immune-suppressive capability of PD-1 (90). Therefore, the usage of Alprazolam may have improved immune response also by inhibiting the suppressive impact of exhaustion markers. Combining Alprazolam with other exhaustion marker inhibitors may further benefit the cause of reducing the size of HIV-1 latency reservoir.

Chronic inflammation caused by HIV-1 infection affects the CNS as well. Neuroinflammation is a major factor of several neurodegenerative disorders including neuroHIV-1 (91–95). Neuroinflammation can contribute to neuronal and immune damage through the chronic activation of microglia and perivascular macrophages of the CNS (15, 35–38). Myeloid cells are long-lived and resistant to cell death caused by infection (35, 37), making them a platform to drive neuroinflammatory events in subjects on suppressive ART therapy. Due to the prolonged life span for patients on ART the prevalence of neurologic issues has increased. Engagement of benzodiazepine receptors, such as those found on microglia and astrocytes, is associated with decreased cytotoxicity (96). Modulation of STAT5 activity secondary to RUNX1 inhibition by Alprazolam was shown in U87MG, an astrocyte cell line, and may be a strategy for addressing this chronic neuroinflammatory state. HIV-1 infected individuals are more likely to be prescribed BDZs than the general population (97, 98), BDZ use is associated with the risk of HIV-1-1 infection (99, 100) and BDZs are a commonly abused substance (101). Given the overlap of substance use and HIV-1 infection, combined with BDZ prescribing practices, it is critical to understand how these drugs may be altering both epigenetics and immune cell function.

## Method

### Ligand Docking

Compounds were built and energy-minimized using the MM2 force field (ChemBio3D Ultra 13.0) with RMS gradient of 0.01 and number of alterations of 104. The minimized structures were then saved as pdb files for the docking simulations. Autodock tools graphical interface was then used to prepare the ligands for docking (102, 103).

The protein structures (RUNX1 unbound, pdb: 1EAN and liganded RUNX1, pdb: 3WTS) (62) were downloaded from the protein data bank then prepared by Autodock tools graphical interface (MGtools 1.5.6rc3) where nonpolar hydrogens were merged, Kollman charges were added, and Gasteiger charges were calculated. The grid box for the docking search was set to include the whole protein structure for the docking search.

AutoGrid 4.2 algorithm (102, 103) was used to evaluate the binding energies between the ligands and the protein and to generate the energy maps for the docking run. For high accuracy mode, the maximum number of evaluations (25 × 106) were used. hundred runs were generated for each ligand by using Autodock 4.2 Lamarckian genetic algorithm for the searches. Cluster analysis was performed on docked results, with a root-mean-square tolerance of 0.5 Å. And the lowest energy conformer from the highest populated cluster was selected as a binding pose for each ligand. The fitting of each pose was independently corrected and validated using POSIT (OpenEye Scientific Software, Santa Fe, NM. http://www.eyesopen.com).

### Cell Culture, Treatments, and Transfections

J-LTR-GFP (Jurkat LTR-GFP) and Jurkat cells were propagated in Roswell Memorial Park Institute (RPMI) media supplemented with 10% Fetal Bovine Serum (FBS), 100 U/mL penicillin, 100 ug/mL streptomycin, and 0.3 mg/mL L-glutamine. J-LTR-GFP cells are Jurkat T-cell based reporter cells that contain an integrated HIV-1 LTR driven GFP and were obtained through the NIH AIDS Reagent Program, Division of AIDS, NIAID, NIH (#11587) from Dr. Olaf Kutsch.

TZM-bl cells (NIH-AIDS Reagent Program, catalog number 8129, NIH, Bethesda, MD) and 293T cells (ATCC) were maintained in Dulbecco's modified Eagle's medium (DMEM; HyClone, GE Healthcare Life Sciences, Chicago, IL) supplemented with 10% FBS, 100 units ml−1 penicillin,100 μg ml−1 streptomycin and 0.3 mg ml−1 L-glutamine (PSG, Gibco, Thermo Fisher Scientific

To transfect 293T with IL17-luciferase reporter, Lipofectamine LTX (Invitrogen) was used according to the manufacturer's instructions.

All cells were grown at 37°C with 5% CO2.

FDA approved BDZs were obtained from (Sigma-Aldrich): Alprazolam (A0357000), clonazepam (C1277. SAHA (SML0061).

### Luciferase Assay

For the determination of HIV-1 LTR reactivation in 293T cells cultures were treated with BDZs or SAHA for 48 hours. Following treatment, cell lysates were prepared using GloLysis buffer (Promega) and luciferase activity was determined using BrightGlo Luciferase Reagent (Promega) and read on a spectrophotometer following manufacturer's instructions. For the determination of IL17 promoter activity, 15,000 293T cells were seeded in each well of a 96 well-plate and transfected 24 h later with a plasmid containing the IL17 promoter controlling the expression of firefly luciferase. Twenty-four h post-transfection cells were treated with BDZs and SAHA. Twenty-four h post-treatment cell lysates were prepared using GloLysis buffer (Promega) and luciferase activity was determined using BrightGlo Luciferase Reagent (Promega) and read on a spectrophotometer following manufacturer's instructions: Dual-Glo Luciferase Assay System (Promega, E2920).

Measurement of RUNX responsive genes. PBMCs from three HIV-1-1 patients who had been suppressed on therapy for <6 months were generously provided by Dr. Frank Maldarelli from the NIH. 10 × 106 PBMCs were divided between three conditions: DMSO control, 50μM Ro5-3335, and 10 μM Alprazolam. PBMCs were cultured in RPMI with 10% FBS and the indicated drugs for 24 h. RNA was extracted from the cells and used for RT-qPCR to detect HIV-1 Gag mRNA. RNA was extracted using Trizol reagent (Invitrogen) following the manufacturer's protocol. Following reverse transcription, the samples were diluted 1:50, and 2.5 microliters were used for quantitative PCR in a BioRad CFX384 qPCR machine. All mRNA analyses were normalized to GAPDH. Nucleic acid amplification was tracked by the SYBR Green method.

The following primer pairs were used for detection:

T-bet 5′GGTTGGAGGACACCGACTAA, 5′ATCCTTCTTGAGCCCCACTT,

IL-2 5′AAACTCACCAGGATGCTCAC, 5′GTCCCTGGGTCTTAAGTGAAAG,

APOBEC3G 5′CCGAGGACCCGAAGGTTAC, 5′TCCAACAGTGCTGAAATTCG,

APOBEC3C 5′AGCGCTTCAGAAAAGAGTGG, 5′AAGTTTCGTTCCGATCGTTG,

IL-7R 5′CCCTCGTGGAGGTAAAGTGC, 5′CCTTCCCGATAGACGACACTC,

GAPDH 5′GCTCACTGGCATGGCCTTCCGTGT, 5′TGGAGGAGTGGGTGTCGCTGTTGA.

### Replication Assay

JLTRG cells were transfected with 1ug of the indicated pNL4-3 molecular clones per 1 × 106 cells. Briefly, cells were incubated with DNA in 0.7ug/ml DEAE-Dextran for 15min at 37C. 1 × STBS was added and cell pellets were resuspended in RPMI-10 in a 24-well plate. Cells were collected every 2 to 3 days, fixed in 1% formaldehyde, and analyzed for %GFP positive cells by flow cytometry.

Generation of RUNX1 binding site mutant virus. A plasmid that contains HIV-1 LTR with mutation in nef gene that does not express nef protein (p398.6) was used as a shuttle vector (104, 105). Site-directed mutagenesis was performed to alter the sequence of the promoter at the 3′ U3 region on the Shuttle vector to obtain mutated binding site for RUNX1. The mutated plasmid was transformed into *E. coli* and selected clones were sent for sequencing. Positive clones were defined as p398.6 mutant RUNX1 binding site (p398.6 mutRUNX BS). Then, to clone the mutant promoter back into pNL4-3 (the entire proviral HIV-1 plasmid), restriction enzymes (NcoI and BamHI) were used to cut the altered sequence from the shuttle vector and pNL4-3 was also cut with the same restriction enzymes. Both plasmid fragments were gel extracted and ligated back into the pNL4-3 vector with T4 ligase. The mutant plasmid was transformed into E. coli and selected colonies were verified by sequencing. A new RUNX1 mutant binding site proviral plasmid (HIV-1Δnef mutRUNX BS) was successfully constructed.

Primers for site-directed mutagenesis:

RUNXMut Fwd 5′atccttgatctgtggatctcacacacacaaggctacttcc

RUNXMut Rev 5′ggaagtagccttgtgtgtgtgagatccacagatcaaggat.

#### Competition Assay

ΔRUNX and Δnef control was used to infect J-LTR-GFP with an equal ratio of infectious particles. Viral stocks were generated by transfection of HEK 293T cells with the pNL4-3 mutants described above. Forty-eight h post-transfection supernatant was harvested and filtered through a 0.2μM filter. Viral stocks were quantified by p24 ELISA and TZMbl beta-galactosidase assay to determine the Gag concentration and infectious units/ml of stock, respectively. The virus was were washed from cells after 24 h and viral growth was tested over 14 days. The peak day is assessed by measuring GFP percentage every other day. At the peak day, the sample taken from the supernatant was used to extract RNA from using Trizol, and 1ml of the supernatant containing virus was used to infect non-infected JLTR-G cells to start the second round of infection. The second round of infection is also assessed for 14 days. Then at the peak day, a sample is taken from the supernatant for RNA extraction and 1ml from the supernatant is used to infect non-infected JLTR-G to start the third round of infection. The peak day is also assessed at the third round of infection to take a sample for RNA extraction. Reverse transcription (RT) reaction was done for each round of infection and then PCR was performed using primers that were designed to amplify the region that flanks the RUNX1 binding site. After extracting the PCR product, TA cloning was performed by inserting the PCR product in a TA vector. Then after bacterial transformation, white clones (which were not stained with X-gal substrate) were chosen to send to sequencing At least 75 clones from each round of infection were sent to be sequenced. Sequence results were analyzed using Clustal W multiple alignments to quantify and compare clones having ΔRUNX vs. Δnef control.

Primers for sequencing of

For 5′TTCAGCTACCACCGCTTGAG

Rev 5′ GTACTCCGGATGCAGCTCTC.

#### ChIP

For ChIP analysis, TZM-bl or PBMC were treated with different treatments and fixed for chip according to manufacturer's protocol: PierceTM Agarose ChIP kit (ThermoFisher, catalog number 26156). All antibodies used were diluted 1:100. Histone H3 (acetyl K9) antibody [AH3-120]-ChIP Grade (Abcam, ab12179). Pierce p300/CBP (CREB-Binding Protein) antibody (NM11) (Thermo scientific, MA5-13634). Human/Mouse STAT5a/b Pan Specific Antibody (R&D Systems, AF2168).

Primers against HIV-1 U3 LTR were used for qPCR:

F 5′CTAGCATTTCGTCACATGGCCCGAGA3′

R 5′GTGGGTTCCCTAGTTAGCCAGAG 3′.

### P24 ELISA

P24 ELISA was performed using the ZeptoMetrix HIV-1 P24 Antigen Elisa Kit using the vendor suggested protocol.

### HEK-BLUE IL-2 Assay

HEK-BLUE IL-2 cells were acquired from Invitrogen (HKB-iL2). Growth Media: DMEM, 4.5 g/l glucose, 2 mM L-glutamine, 10% (v/v) fetal bovine serum (FBS), 100 U/ml penicillin, 100 mg/ml streptomycin,100 mg/ml Normocin. HEK-BLUE IL-2 cells were generated by transfecting HEK293 cells with the human IL-2Rα, IL-2Rβ, and IL-2Rγ genes, along with the human JAK3 and STAT5 genes to obtain a fully active IL-2 signaling pathway as well as a reporter gene expressing a secreted embryonic alkaline phosphatase (SEAP) under the control of the IFN-b minimal promoter fused to four STAT5 binding sites. HEK BLUE IL-2 Cells were maintained in growth media and detached from the culture flask through gentle washes using PBS. Cells were seeded in 96 well-plates and treated with appropriate drugs and then incubated in a 37C incubator for 24 or 48 h before cell suspension is harvested for SEAP quantification using QUANTI-BLUE, which is a solution that changes the cell suspension color from pink to blue in the presence of alkaline phosphatase.

Intracellular cytokine staining. PBMCs from HIV-1 positive individuals suppressed on therapy for >6 months were thawed, resuspended in 1 × 106 per ml in RPMI/IL2 (RPMI + 10% FBS, Penn/Strep, L-glutamine, 30 IU/ml IL-2), had 1 ml of cell suspension placed in a 12 × 75 mm falcon tube per condition and allowed to rest overnight at 37C with 5% CO2. SAHA, Alprazolam, or vehicle control were added to the tubes as appropriate and incubated for 4 h. After incubation with drugs, anti-CD29 and anti-49d co-stimulatory antibodies (BD Biosciences) were added along with pooled HIV-1-1 B Gag peptides (AIDS Reagent) at 2ug/ml final concentration per peptide. One h after the addition of peptides BD Golgi Stop was added and PBMCs were incubated overnight at 37C with 5% CO2. PBMCs were then stained for CD3, CD4, CD8, and viability (Zombie Yellow, BioLegend) before being permeabilized with BD Cytofix/Cytoperm reagent. Permeabilized cells were stained for IL-2 and IFNγ before being fixed and analyzed by flow cytometry.

## Ethical Statement

PBMCs for intracellular cytokine staining were obtained from venous blood draw of HIV-1 positive individuals approved under the University of the Sciences' protocol (IRB protocol 900702-3 and 797649-3). Blood draws were performed at the Smith Center for Urban Health and Infectious Disease, East Orange, NJ, and written informed consent was obtained for the collection of blood donations from participating subjects.

PBMCs for the analysis of RUNX1 responsive genes were obtained from Dr. Frank Maldarelli and the NIH Clinical Center. The human sample collection protocol was approved by the NIH Clinical Center Institutional Review Board as part of a separate ongoing study. Written informed consent was obtained in all cases and all applicable protections of patient rights and privacy applied. For this study, specific samples were requested from the sample bank based on the given criteria.

## Data Availability Statement

The original contributions presented in the study are included in the article/Supplementary Material, further inquiries can be directed to the corresponding authors.

## Ethics Statement

The studies involving human participants were reviewed and approved by University of the Sciences Review Board (IRB protocol 900702-3 and 467 797649-3). The patients/participants provided their written informed consent to participate in this study.

## Author Contributions

AL, WE, and AA performed experiments, analyzed data, and designed experiments. AS and RV aided in experiments. AL and ZK wrote the manuscript. SC oversaw and designed the docking experiments and aided in writing the manuscript. ZK oversaw and aided in the design of the experiments and performed analysis. RV aided in experimental design and manuscript writing. All authors contributed to the article and approved the submitted version.

## Conflict of Interest

The authors declare that the research was conducted in the absence of any commercial or financial relationships that could be construed as a potential conflict of interest.
