# Supplementary material for: Alprazolam Prompts HIV-1 Transcriptional Reactivation and Enhances CTL Response Through RUNX1 Inhibition and STAT5 Activation
Source: Front Neurol. 2021 Jul 22;12:663793. doi: 10.3389/fneur.2021.663793 (PMC8339301; doi:10.3389/fneur.2021.663793)
Supplement: Supplementary file 1 [file Data_Sheet_1.pdf]

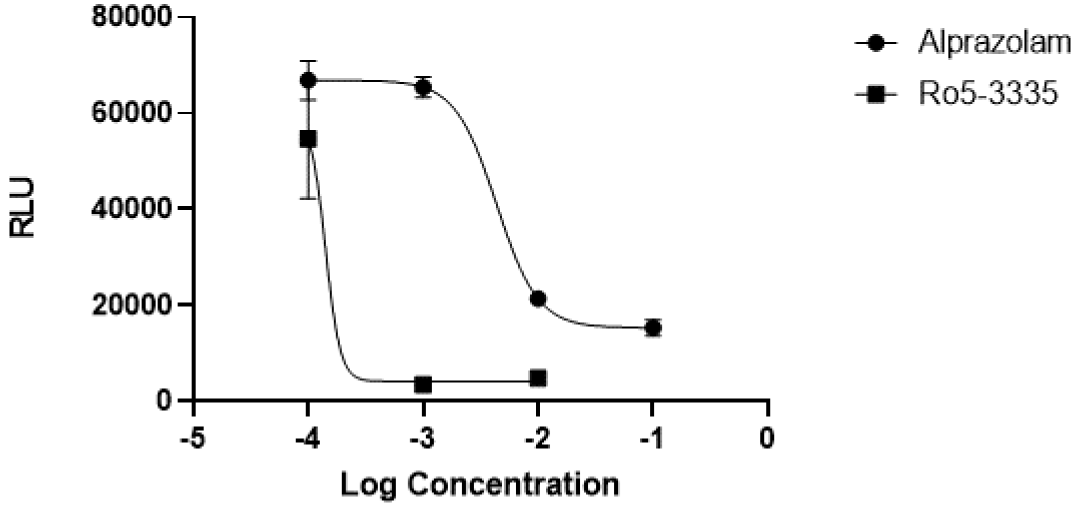

S1. RUNX1 inhibition IC50 analysis.293T cells were transfected with IL17 promoter driven luciferase reporter plasmid and treated with Alprazolam and R05-3335 from 1nM to 1µM and luciferase was measured.

Supplemental 1

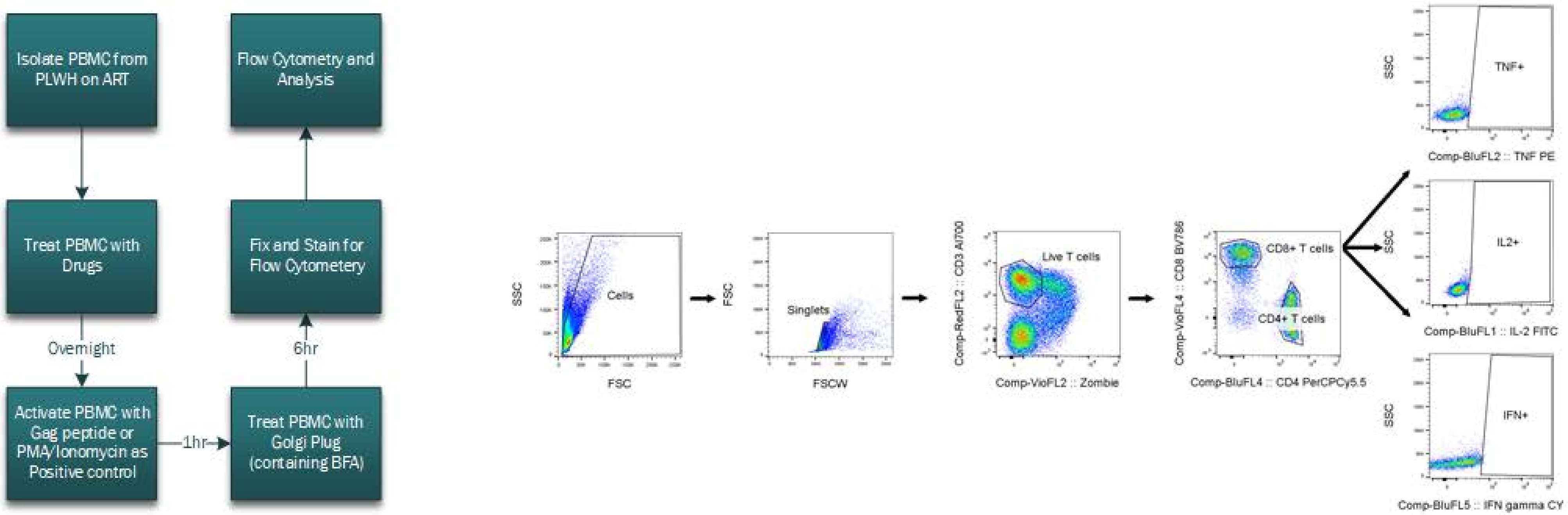

S2. Example of gating for intracellular cytokines in CD8+ T cells. PBMCs from HIV-1 infected subjects were treated and activated using gag pool peptides and protein export was blocked using Golgi plug. Cells were stained for live/dead, CD3, CD4, CD8, and intracellular cytokines.

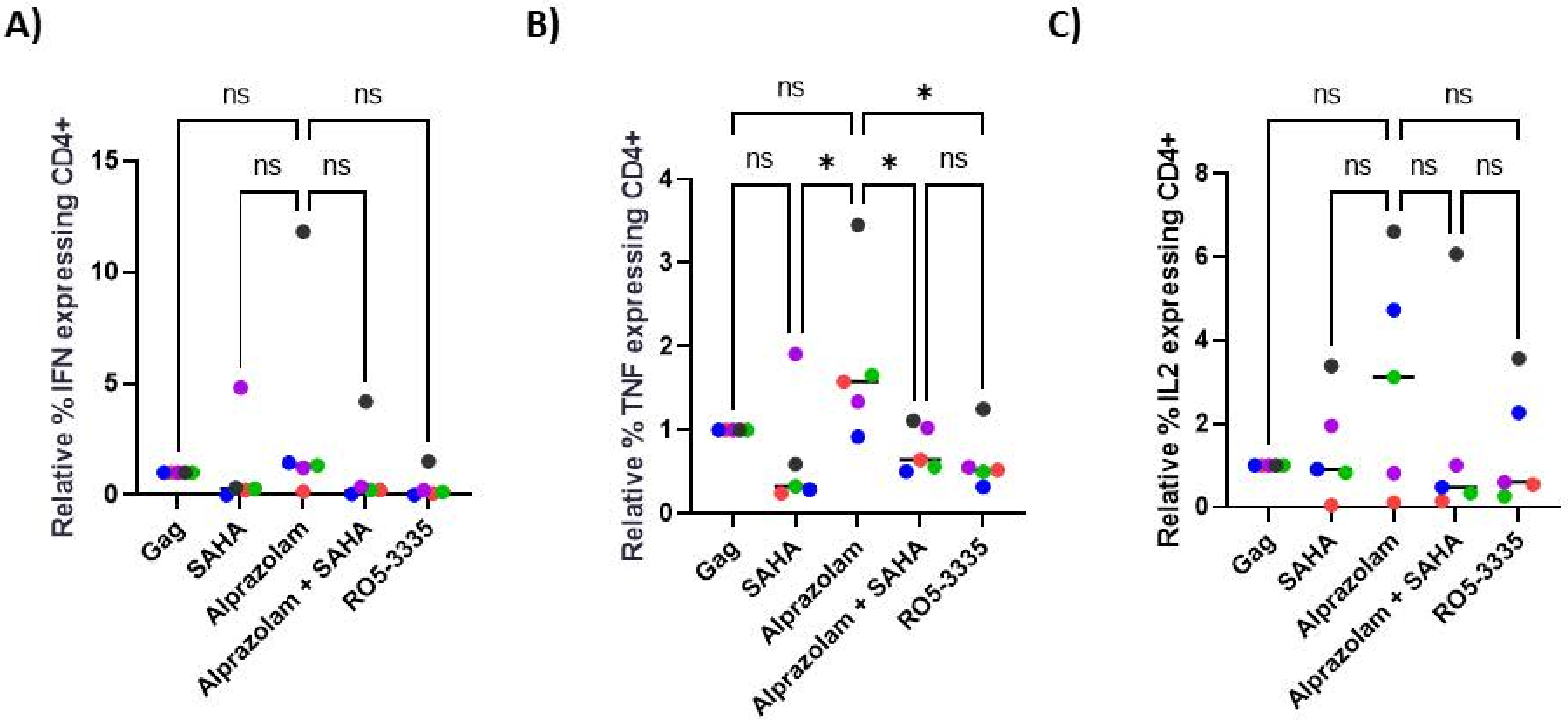

Figure S3. The effect of alprazolam on cytokine production in CD4+ T cells. PBMCs from 5 HIV-1 positive individuals suppressed on therapy were treated with vehicle control, 500 nM SAHA, 100 nM alprazolam , SAHA plus alprazolam or 100nM Ro5-3335 overnight and then exposed to a set overlapping Gag peptides in the presence of Brefaldin A for 6 hours. Cells were stained for live/dead marker, CD3, CD4, CD8 and cytokines of interest. The number of A) IFNγ, B) TNFα C) IL-2 expressing CD4+ T-cells for each condition is shown normalized to untreated control.
